# Monomer DJ-1 and Its N-Terminal Sequence Are Necessary for Mitochondrial Localization of DJ-1 Mutants

**DOI:** 10.1371/journal.pone.0054087

**Published:** 2013-01-10

**Authors:** Chinatsu Maita, Hiroshi Maita, Sanae M. M. Iguchi-Ariga, Hiroyoshi Ariga

**Affiliations:** 1 Graduate School of Pharmaceutical Sciences, Hokkaido University, Sapporo, Japan; 2 Graduate School of Agriculture, Hokkaido University, Sapporo, Japan; University of Melbourne, Australia

## Abstract

*DJ-1* is a novel oncogene and also a causative gene for familial Parkinson’s disease (*park7*). DJ-1 has multiple functions that include transcriptional regulation, anti-oxidative reaction and chaperone and mitochondrial regulation. Mitochondrial dysfunction is observed in DJ-1-knockout mice and fry, and mitochondrial DJ-1 is more protective against oxidative stress-induced cell death. Although translocation of DJ-1 into mitochondria is enhanced by oxidative stress that leads to oxidation of cysteine 106 (C106) of DJ-1, the characteristics of mitochondrial DJ-1 and the mechanism by which DJ-1 is translocated into mitochondria are poorly understood. In this study, immunostaining, co-immunoprecipitation, cell fractionation and pull-down experiments showed that mutants of glutamine 18 (E18) DJ-1 are localized in mitochondria and do not make homodimers. Likewise, DJ-1 with mutations of two cysteines located in the dimer interface, C46S and C53A, and pathogenic mutants, M26I and L166P DJ-1, were found to be localized in mitochondria and not to make homodimers. Mutant DJ-1 harboring both E18A and C106S, in which C106 is not oxidized, was also localized in mitochondria, indicating that oxidation of C106 is important but not essential for mitochondrial localization of DJ-1. It should be noted that E18A DJ-1 was translocated from mitochondria to the cytoplasm when mitochondrial membrane potential was reduced by treatment of cells with CCCP, an uncoupler of the oxidative phosphorylation system in mitochondria. Furthermore, deletion or substitution of the N-terminal 12 amino acids in DJ-1 resulted in re-localization of E18A, M26I and L166P DJ-1 from mitochondria into the cytoplasm. These findings suggest that a monomer and the N-terminal 12 amino acids are necessary for mitochondrial localization of DJ-1 mutants and that conformation change induced by C106 oxidation or by E18 mutation leads to translocation of DJ-1 into mitochondria.

## Introduction

The *DJ-1* gene has been identified by us to be a novel oncogene that transforms NIH3T3 cells in cooperation with the activated *ras* gene [1] and was later found to be a causative gene for familial Parkinson’s disease (*park7*) [2]. DJ-1 is expressed ubiquitously in cultured cells and tissues and is localized in the cytoplasm, nucleus and mitochondria [1], [3]–[5]. DJ-1 has multiple functions, including transcriptional regulation [6]–[14], anti-oxidative stress function [3], [15]–[19], and chaperone [20], [21], protease [22]–[24] and mitochondrial regulation [25]–[35]. DJ-1 contains three cysteine residues at amino acid numbers 46, 53 and 106 (C46, C53 and C106, respectively). Of those cysteines, C106 is highly sensitive to oxidative stress and is oxidized in forms of SOH, SO_2_H and SO_3_H [3], [16]. Mutation of C106 results in complete loss of DJ-1′s activity [3], [15], [17], and highly oxidized DJ-1 was found in the brains of patients with Parkinson’s disease and Alzheimer’s disease [36], [37].

Although a portion of DJ-1 is present in mitochondria [4], [28], the degree of translocation of DJ-1 into mitochondria is stimulated by oxidative stress, and oxidation of C106 with SO_2_H is necessary for mitochondrial translocation of DJ-1 [3]. Mitochondria-target sequence-conjugated DJ-1 has been shown to be more protective against oxidative stress-induced cell death [27]. It has been reported that activity of mitochondrial complex I is decreased in patients with Parkinson’s disease [38]–[42] and that mitochondrial dysfunctions occur in DJ-1 knockout mice and fry [33], [43]. DJ-1 binds to subunits of mitochondrial complex I and regulates its activity [28]. When mitochondrial membrane potential is decreased, DJ-1 is translocated into mitochondria, resulting in induction of mitophagy, which is clearance of damaged mitochondria [29], [31], [34]. These findings suggest that DJ-1 plays a role in homeostasis of mitochondria. Since DJ-1 has no mitochondrial target sequence, the precise mechanism by which DJ-1 is translocated into mitochondria is still not known. DJ-1 binds to several chaperones, including Hsp70, CHIP and mitochondrial Hsp70/Mortarin/Grp75, suggesting that translocation of DJ-1 into mitochondria is associated with other proteins, including mitochondrial Hsp70 [26].

In this study, we found that DJ-1 with mutation at glutamine 18 (E18) is localized in mitochondria and does not form a homodimer. Likewise, dimer formation-negative DJ-1 mutants, including pathogenic M26I and L166P DJ-1, are also localized in mitochondria, indicating that monomer DJ-1 is localized in mitochondria. Furthermore, we found that the N-terminal 12 amino acids in DJ-1 are necessary for mitochondrial translocation of DJ-1.

## Materials and Methods

### Cells

HeLa and 293T cells were purchased from American Tissue culture collection (ATCC). DJ-1-knockout (DJ-1(−/−)) and its parental DJ-1(+/+) mouse cells that had been immortalized with SV40 T-antigen were described previously [44]. The cells were cultured in Dulbecco’s modified Eagle’s medium (DMEM) with 10% calf serum. DJ-1(−/−) cells were transfected with expression vectors for human wild-type, C106S and E18A DJ-1-HA together with that for the hygromycin B-resistant gene and cultured in the presence of 400 µg/ml hygromycin B. About 3–4 weeks after transfection, hygromycin B-resistant cells were selected and named WT-HA, C106S-HA and E18A-HA cells, respectively.

### Western Blotting and Antibodies

To examine the expression levels of endogenous proteins or proteins attached with various tags in cells, proteins were extracted from cells with a buffer containing 150 mM NaCl, 1 mM EDTA, 20 mM Tris (pH 8.0) and 0.5% NP-40. Proteins were then separated on a 12% polyacrylamide gel and subjected to Western blotting with respective antibodies. In the case of treatment of cells with disuccinimidyl suberate (DSS), above buffer containing 1% NP40 was used. Proteins on the membrane were reacted with an IRDye 800- (Rockland, Philadelphia, PA, USA) or Alexa Fluor 680-conjugated secondary antibody (Molecular Probes, Eugene, OR, USA) and visualized by using an infrared imaging system (Odyssey, LI-COR, Lincoln, NE, USA). The antibodies used were anti-HA (1∶1000, Santa Cruz Biotechnology, Santa Cruz, CA, USA), anti-FLAG (1∶1000, M2, Sigma, St. Louis, MO USA), anti-OxPhos complex V (1∶1000, Molecular Probes), anti-lamin B (1∶200, C-20, Santa Cruz), anti-GAPDH (1∶4000, Chemicon, Temecula, CA, USA) and rat anti-DJ-1 (1∶100) antibodies. The rat anti-DJ-1 monoclonal antibody was established by us after immunization of rats with recombinant human DJ-1. After proteins on membranes had been reacted with Alexa Fluor 680-conjugated anti-mouse, rabbit, rat or goat antibody (Molecular Probes, Eugene, OR) or IRDye 800-conjugated anti-mouse or rabbit antibody (Rockland, Philadelphia, PA), the proteins were visualized by using an infrared imaging system (Odyssey, LI-COR, Lincoln, NE).

### Co-immunoprecipitation Assay

293T cells in 6-cm dishes were transfected with 3 µg of expression vectors for FLAG-tagged wild-type or various mutant DJ-1s together with those for corresponding One-STrEP-tagged DJ-1 by the calcium phosphate precipitation technique. Fifty-four hrs after transfection, proteins were extracted from cultured cells as described in the Western blotting and antibodies section. One percent of NP40 was used instead of 0.5% NP40. Proteins were immunoprecipitated with an agarose-conjugated anti-FLAG antibody (1∶500, M2, Sigma) and precipitates were analyzed by Western blotting with an anti-STrEP antibody (1∶1500, IBA, Göttingen, Germany). Proteins on membranes were visualized as described above.

### Pull-down Assays

DJ-1(−/−) cells in 6-cm dishes were transfected with 4 µg of expression vectors for HA-tagged wild-type DJ-1 or various mutant DJ-1s together with those for corresponding One-STrEP-tagged DJ-1 by Lipofectamine 2000 (Invitrogen, Carlsbad, CA, USA) according to a manufacturer’s protocol. Twenty-six hrs after transfection, proteins were extracted from transfected cells as described above and applied onto Strep-Tactin Sepharose (ABI). Proteins bound to Strep-Tactin Sepharose were subjected to Western blotting with anti-HA (1∶1000, Santa Cruz) and anti-StrEP (1∶1500, IBA) antibodies.

### Indirect Immunofluorescence

DJ-1(−/−) or HeLa cells in 6-well plates were transfected with 4 µg of expression vectors for HA-tagged wild-type DJ-1 or various DJ-1 mutants by lipofectamine 2000. Twenty-four hrs after transfection, 100 nM MitoTracker Red CMXRos (Molecular Probes) was added to the cells and the cells were cultured for another 45 min. After the cells had been washed and cultured in the normal medium for 2 hrs, they were fixed with 4% paraformaldehyde for 15 min and then with 3∶7 ratio of acetone/methanol for 5 min or with 0.1–0.2% Triton X-100 for 10 min and reacted with an anti-HA antibody (1∶100, Santa Cruz) for 1 hr. The cells were then reacted with an FITC-conjugated anti-rabbit IgG, and their nuclei were stained with DAPI. The cells were then observed under a fluorescent microscope (Biorevo BZ-9000, Keyence, Osaka, Japan) or confocal laser fluorescent microscope (LSM510 META, Carl Zeiss, Jena, Germany).

### Isoelectric Focusing

GST-free wild-type DJ-1 and mutants of DJ-1 were prepared as described above and reacted with 1 M H_2_O_2_ for 60 min at room temperature. After DJ-1 had been precipitated with acetone, it was separated on pH 2.5–8 ranges of an isoelectric focusing gel, transferred onto nitrocellulose membranes and reacted with anti-DJ-1 antibodies (1∶4000, rabbit polyclonal antibody or 1∶1000, mouse monoclonal antibody, clone 3E8, MBL).

### Subcellular Fractionation

DJ-1(+/+) or DJ-1(−/−) cells harboring HA-tagged wild-type or various mutant DJ-1s were washed twice with cold PBS and fractionated by using a Protein extract subcellular proteome extraction kit (Calbiochem/Merk, Darmstadt, Germany) according to the manufacturer’s protocol.

### Ethics Statement

All animal experiments were carried out in accordance with the National Institutes of Health Guide for the Care and Use of Laboratory Animals, and the protocols were approved by the Committee for Animal Research at Hokkaido University (the permit number 08-0467).

## Results

### E18 Mutants of DJ-1 do not form a Homodimer

**Figure 1:**
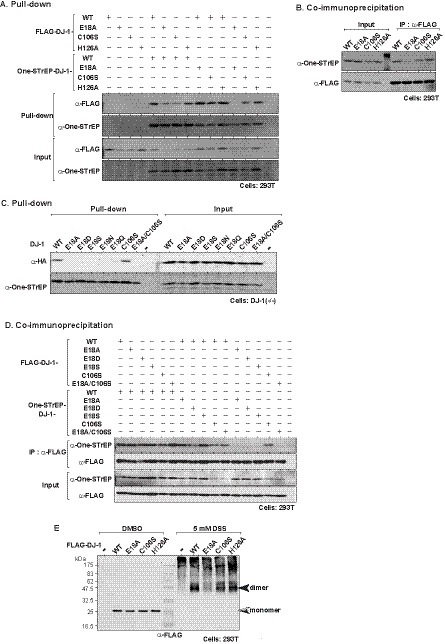
Dimer formation of wild-type and various mutants of DJ-1. A and C. FLAG-tagged wild-type DJ-1 and various mutants of DJ-1 were transfected into 293T cells (A) and DJ-1(−/−) cells (C) together with One-STrEP-tagged wild-type DJ-1 and various mutants of DJ-1. Proteins extracted from transfected cells were applied to Strep-Tactin Sepharose. Proteins bound to Strep-Tactin Sepharose were subjected to Western blotting with anti-HA and anti-StrEP antibodies as described in Materials and methods. B and D. FLAG-tagged wild-type DJ-1 and various mutants of DJ-1 were transfected into 293T cells. At 54 hrs after transfection, proteins extracted from transfected cells were immunoprecipitated with an anti-FLAG M2 agarose and precipitates were analyzed by Western blotting with an anti-StrEP antibody as described in Materials and methods. E. 293T cells were transfected with FLAG-tagged wild-type DJ-1 and various mutants of DJ-1. At 24 hrs after transfection, proteins extracted from transfected cells were treated with 5 mM DSS for 30 min and subjected to Western blotting with an anti-FLAG antibody.

Homodimer formation is necessary for DJ-1 to elicit its functions, and L166P DJ-1 found in patients with Parkinson’s disease lacks dimer-forming activity, resulting in loss of function [2]. Since the crystal structure of mutant DJ-1 at glutamine at an amino acid number 18, E18A DJ-1, is similar to that of wild-type DJ-1 ([45], [46] our unpublished data), it is thought that E18A DJ-1 also makes a homodimer. To examine dimer formation of various DJ-1 mutants, including E18A DJ-1, 293T cells were transfected with FLAG-tagged wild-type, E18A, C106S and H126A DJ-1 together with various combinations of One-STrEP-tagged DJ-1. Proteins extracted from transfected cells were applied onto Strep-Tactin sepharose, and proteins bound to sepharose were analyzed by Western blotting with anti-STrEP and anti-FLAG antibodies. The results showed that C106S, H126A and E18A DJ-1 all made heterodimers with wild-type DJ-1 but that homodimer formation occurred in wild-type, C106S and H126A DJ-1 but not in E18A DJ-1 (Fig. 1A). No or reduced homodimer formation of E18A DJ-1 was further confirmed by co-immunoprecipitation experiments, in which 293T cells were transfected with FLAG and One-STrEP-tagged DJ-1 as described above. After transfection, proteins extracted from transfected cells were immunoprecipitated with an anti-FLAG antibody and the precipitates were analyzed by Western blotting with anti-STrEP and anti-FLAG antibodies. The results showed that while One-STrEP-tagged wild-type, C106S and H126A DJ-1 were well-precipitated with the anti-FLAG antibody, only a small amount of One-STrEP-tagged E18A DJ-1 was co-immunoprecipitated (Fig. 1B). Furthermore, homodimer formation of other E18 mutants of DJ-1 and E18A/C106S double mutant of DJ-1 was examined by pull-down experiments as described in the legend of Fig. 1A, in which DJ-1(−/−) cells were used instead of 293T cells to rule out the effect of endogenously expressed DJ-1 in cells. As shown in Fig. 1C, all of the E18 mutants of DJ-1 lost dimer-forming activity. While C106S DJ-1 formed dimer, E18A/C106S DJ-1 also lost dimer-forming activity. No or reduced homodimer formation of other E18 mutants of DJ-1 and E18A/C106S double mutant of DJ-1 1 was further confirmed by co-immunoprecipitation experiments after 293T cells had been transfected with E18 mutants of DJ-1 and E18A/C106S DJ-1 (Fig. 1D). We then used cross-linking experiments to confirm reduced homodimer-forming activity of E18A DJ-1. 293T cells were transfected with FLAG-tagged wild-type, E18A, C106S and H126A DJ-1. At 24 hrs after transfection, proteins extracted from transfected cells were reacted with 5 mM disuccinimidyl suberate (DSS) for 30 min and analyzed by Western blotting with the anti-FLAG antibody. Molecular mass of dimer DJ-1 is around 45 kDa. As shown in Fig. 1E, the dimer level of FLAG-E18A DJ-1 was lower than that of wild-type, C106S and H126A DJ-1 even under the condition of 5 mM DSS. These results clearly indicate that E18 is essential for DJ-1 to make a homodimer.

### E18 Mutants and E18A/C106S Double Mutant of DJ-1 are Localized in Mitochondria

**Figure 2:**
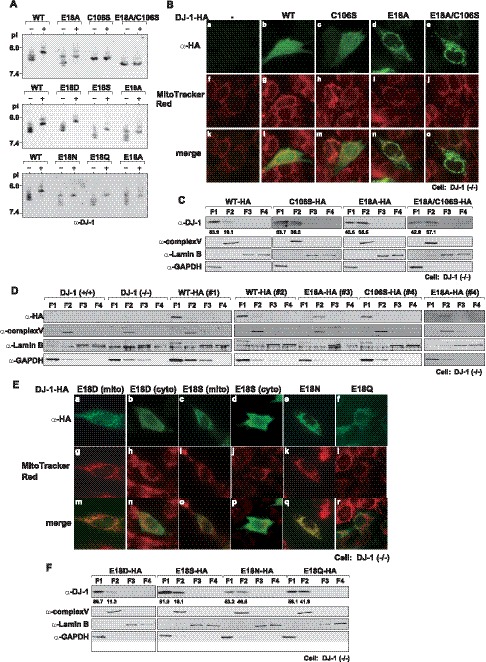
Localization of monomer-forming mutants of DJ-1. A. GST-free DJ-1s were expressed in and purified from *E. coli* and GST-free DJ-1s were prepared. GST-free DJ-1s were reacted with 1 M H_2_O_2_ for 60 min at room temperature. After DJ-1 had been precipitated with acetone, it was separated on pH 2.5–8 ranges of an isoelectric focusing gel, followed by Western blotting with anti-DJ-1 antibodies as described in Materials and methods. B and E. HA-tagged wild-type DJ-1 and various mutants of DJ-1 were transfected into DJ-1(−/−) cells. At 24 hrs after transfection, mitochondria in cells were stained with MitoTracker Red. The cells were then fixed with 0.2% triton X-100 (B) or with 4% paraformaldehyde and with acetone/methanol (E) and reacted with an anti-HA antibody. After cells had been reacted with an FITC-conjugated secondary antibody, proteins were visualized as described in Materials and methods. C and F. DJ-1(−/−) cells that had been transfected as described in the legends for Figures 2B and 2E were fractionated by using a subcellular fractionation kit. Proteins in each fraction were analyzed by Western blotting with anti-HA (Figure 2C), anti-DJ-1 (Figure 2F), anti-OxPhos complex V (Complex V), anti-Lamine B and anti-GAPDH antibodies as described in Materials and methods. Fractions 1 (F1), 2 (F2), 3 (F3) and 4 (F4) contain the cytosol, membrane-containing organelle, nucleus and cytoskeleton, respectively. D. DJ-1(−/−) cells expressing HA-tagged wild-type and various mutants of DJ-1 were fractionated by using a subcellular fractionation kit. Proteins in each fraction were analyzed by Western blotting as described in the legend for Figure 2C.

**Figure 3:**
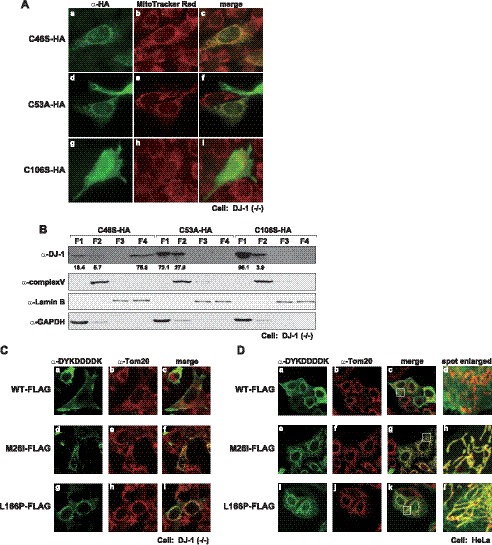
Localization of dimer formation-negative and pathogenic mutants of DJ-1. A. DJ-1(−/−) cells were transfected with HA-tagged wild-type, C53A and C106S DJ-1. At 25 hrs after transfection, mitochondria in cells were stained with MitoTracker Red. The cells were then fixed with 4% paraformaldehyde and with 0.2% triton X-100 and reacted with an anti-HA antibody. After cells had been reacted with an FITC-conjugated secondary antibody, proteins were visualized by using a confocal laser microscope as described in Materials and methods. B. DJ-1(−/−) cells that had been transfected as described in the legends for Figure 3A were fractionated by using a subcellular fractionation kit. Proteins in each fraction were analyzed by Western blotting as described in the legend for Figure 2C. C and D. DJ-1(−/−) cells (C) and HeLa cells (D) were transfected with FLAG-tagged wild-type, M26I and L166P DJ-1, and localization of proteins was examined as described in the legend of Figure 3A.

**Figure 4:**
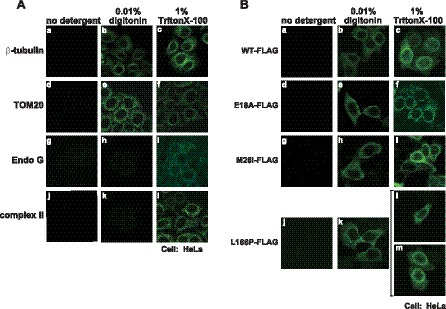
Identification of the mitochondrial region of DJ-1 mutants. HeLa cells were transfected with FLAG-tagged wild-type, E18A, M26I and L166P DJ-1. At 24 hrs after transfection, cells were fixed with 4% paraformaldehyde, permeabilized with 0.01% digitonin for 5 min or with 1% TritonX-100 for 10 min, and subjected to immunostaining as described in Materials and methods. Cell images were observed using a confocal laser microscope. A. The cells were reacted with anti-β-tubulin, anti-TOM20, anti-Endo G and anti-70-kDa subunit of complex II antibodies. B. The cells were reacted with an anti-FLAG antibody.

It has been reported that some E18 mutants of DJ-1 are localized in mitochondria [45] and that translocation of DJ-1 into mitochondria requires oxidation of C106 [3]. Oxidative status of various E18 mutants after exposure to H_2_O_2_ was examined by isoelectric focusing gels. The results showed that wild-type and all of the E18 mutants of DJ-1 were highly oxidized after exposure to H_2_O_2_ but that C106S and E18A/C106S DJ-1 were less oxidized or not oxidized (Fig. 2A). This result and the result in Fig. 1C showed that oxidative status of C106S DJ-1 was not changed by additional mutation of E18, but E18A/C106S DJ-1 did not form a homodimer. To examine the relationship between oxidative status and property of DJ-1, localization of these mutants of DJ-1 were examined. DJ-1(−/−) cells were transfected with HA-tagged wild-type, C106S, E18A and E81A/106S DJ-1, fixed with 0.2% triton X-100 and reacted with an anti-HA antibody followed by an FITC-conjugated secondary antibody. Mitochondria were stained with MitoTracker Red, and FITC (green) and MitoTracker Red (red) colors were merged. The results showed that wild-type and C106S DJ-1 were localized mainly in the cytoplasm and in the nucleus and mitochondria to a lesser extent (Fig. 2B-b and 2B-c) and that E18A DJ-1 was localized in mitochondria (Fig. 2B-d). E18A/106S DJ-1 was, on the other hand, found to be localized in mitochondria (Fig. 2B-e). Localization of mutants of DJ-1 in transfected DJ-1(−/−) cells was also examined by a subcellular fractionation study. After fractionation of cells, four fractions, including fraction 1 (cytosol), fraction 2 (membrane-containing organelle), fraction 3 (nucleus) and fraction 4 (cytoskeleton), were obtained, and their proteins were analyzed by Western blotting and proportion of localization of DJ-1 is shown under figures (Fig. 2C). F1F0-ATPase, which is a subunit of mitochondrial complex V, and lamin B were used as marker proteins in the mitochondria and nucleus, respectively. Since GAPDH is preferentially localized in the cytoplasm but partly localized in the nucleus and membrane fractions, GAPDH was also used as a marker protein. The results of Western blotting with an anti-DJ-1 antibody showed that while wild-type DJ-1-HA was localized mainly in the cytosolic fraction (83.9%) and faintly in the membrane-containing organelle (16.1%) and while C106S DJ-1-HA was localized in the cytosolic fraction (63.7%), more than half of E18A DJ-1-HA and E18A/C106S DJ-1-HA were localized in the membrane-containing organelle (56.5 and 57.1%, respectively) (Fig. 3B). Localization of mutants of DJ-1 was also examined using stable cells lines. To do this experiment, DJ-1(−/−) cells were transfected with HA-tagged wild-type, E18A and C106S DJ-1 together with an expression vector for hygromycin B, and hygromycin B-resistant DJ-1(−/−) cells expressing wild-type DJ-1 or mutants of DJ-1 were selected. The results of Western blotting with an anti-HA antibody showed that while wild-type DJ-1-HA was localized mainly in the cytosolic fraction and faintly in the membrane-containing organelle and while C106S DJ-1-HA was preferentially localized in the cytosolic fraction, E18A DJ-1-HA in two cells lines, #3 and #4, was localized only in the membrane-containing organelle (Fig. 2D). Localization of other E18 mutants was then examined as described in the legend of Fig. 2B. E18D-HA and E18S-HA DJ-1 showed two patterns of localization, in the mitochondria (Figs. 2E-a and 2E-c) and cytoplasm (Figs. 2E-b and 2E-d). E18N-HA and E18Q-HA DJ-1, like E18A-HA DJ-1, were localized in mitochondria (Figs. 2E-e and 2E-f). Fractionation studies using these transfected cells also showed mitochondrial localization of E18 mutants (Fig. 2F). Different ratio of localization of DJ-1 in organelle between immunostaining and fractionation studies is thought to be due to a fixation method used in the immunostaining study as later shown in Fig. 4. These results suggest that oxidation of C106 is not essential for mitochondrial localization of DJ-1 and that conformation change induced by C106 oxidation or by E18 mutation leads to mitochondrial localization of DJ-1.

### Monomer DJ-1 is Localized in Mitochondria

E18 mutants of DJ-1 and pathogenic L166P DJ-1 do not form homodimers and are localized in mitochondria (this study and references [3], [45]). We therefore speculated that monomer DJ-1 is localized in mitochondria. Cysteine residues at amino acid numbers 46 and 53 in DJ-1, C46 and C53, are located in the dimer interface [46] and mutations of C46 and C53 reduced anti-oxidative stress activity of DJ-1 [15]. To examine the above possibility, HA-tagged C46A, C53A and C106S DJ-1 were transfected into DJ-1(−/−) cells and their localization was examined by fixation of cells with 0.2% triton X-100 followed by staining with an anti-HA antibody. As shown in Fig. 3A, C46S DJ-1-HA and C53A DJ-1-HA, but not C106S DJ-1-HA, were localized in mitochondria. Cell fractionation studies also showed C46S DJ-1-HA was localized in the cytoskeleton in addition to mitochondria and that about 30% of C53A DJ-1-HA was localized mitochondria. Majority of C106S DJ-1-HA was localized in the cytoplasm (Fig. 3B).

Furthermore, localization of pathogenic mutants of FLAG-tagged M26I and L166P DJ-1 and of FALG-tagged wild-type DJ-1 were examined after transfection of these DJ-1s into DJ-1(−/−) and HeLa cells (Figs. 3C and 3D, respectively). The results showed that M26I DJ-1-FLAG and L166P DJ-1-FLAG were localized in mitochondria and that wild-type DJ-1-FLAG was localized mainly in the cytoplasm in both cell types. Cell fractionation studies also showed that about half of M26I DJ-1-FLAG and L166P DJ-1-FLAG were localized in mitochondria. We have already shown that homodimer-forming activity of M26I DJ-1 is weak compared to that of wild-type DJ-1 [26]. These results suggest that monomer DJ-1 is translocated into mitochondria.

### DJ-1 is Localized Preferentially Inside the Outer Mitochondrial Membrane

There are several reports on the localization of DJ-1 in mitochondria, including the outer membrane [3], inner membrane [28] and matrix [4]. To examine the localization of monomer DJ-1 in mitochondria, two permeabilization conditions were used before reaction of cells with primary antibodies. Digitonin permeabilizes the peripheral membrane, but the mitochondrial outer membrane remains intact. Triton-X100, on the other hand, permeabilizes both peripheral and mitochondrial membranes, resulting in reactivity of proteins localized in the intermembrane space, inner membrane and matrix to respective antibodies. When HeLa cells were treated with 0.01% digitonin, Tom20, which is localized in the mitochondrial outer membrane, was observed, but Endo G, which is localized in the matrix, was not observed (Figs. 4A-g and 4A-h). When HeLa cells were treated with 1% TritonX-100, on the other hand, Tom20, Endo G and 70-kDa subunit of complex II, which is localized in the inner membrane, were observed (Figs. 4A-f, 4A-I and 4A-l).

HeLa cells were transfected with FLAG-tagged wild-type, E18A, M26I and L166P DJ-1, treated with 0.01% digitonin or with 1% TritonX-100, and reacted with an anti-FLAG antibody. Localization of DJ-1-FLAG was then observed by using a confocal laser microscope. Cytoplasmic localization of wild-type DJ-1-FLAG was observed with both digitonin and TritonX-100 treatment, but localization in the nuclear edge became clear with TritonX-100 treatment (Figs. 4B-b and 4B-c). All of the mutants of DJ-1 were weakly stained by digitonin treatment and strongly stained in mitochondria after cells had been treated with TritonX-100, indicating that E18, M26I and L166P DJ-1 were mainly localized inside the mitochondrial outer membrane and that small portions of them were localized in the outer membrane.

### Reduced Mitochondrial Membrane Potential Re-localizes E18 Mutant of DJ-1 from Mitochondria to the Cytoplasm

**Figure 5:**
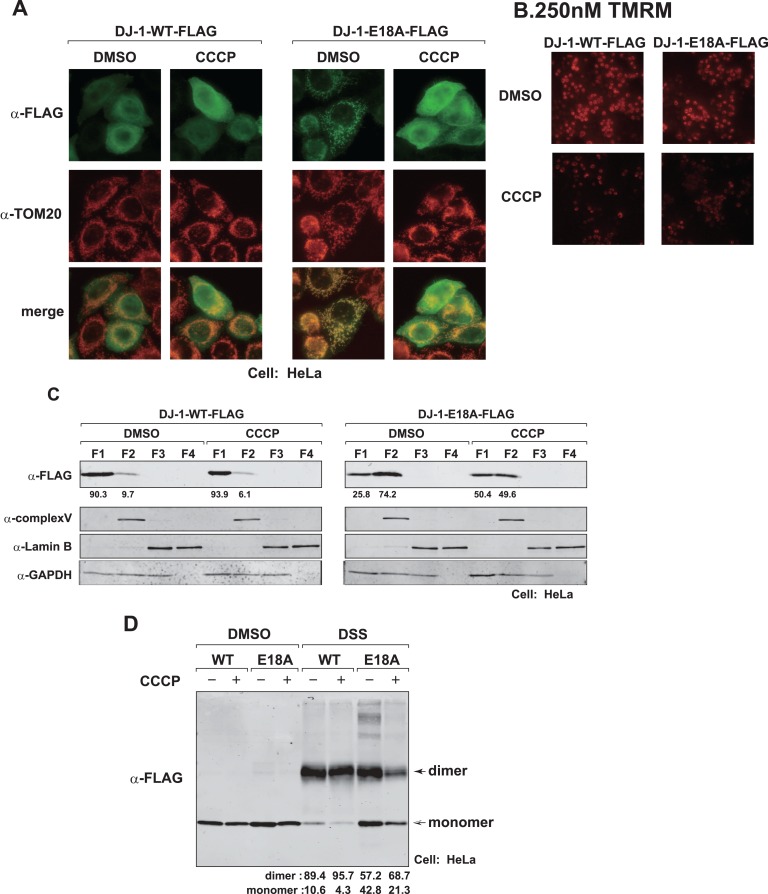
Effect of reduction of mitochondrial membrane potential on localization of E18A DJ-1. A. HeLa cells were transfected with FLAG-tagged wild-type and E18A DJ-1. At 11 hrs after transfection, the cells were treated with 20 µM CCCP for 7 hrs, fixed with 4% paraformaldehyde and with 0.2% TritonX-100, and stained with anti-FLAG and anti-Tom20 antibodies. Cell images were then observed under a fluorescent microscope. B. To examine mitochondrial membrane potential, transfected HeLa cells described above were reacted with 250 µM TMRM for 15 min at 1 hr after CCCP addition, and fluorescence images of TMRM were observed under a fluorescent microscope. C. HeLa cells that had been transfected as described in the legends for Figure 5A were fractionated by using a subcellular fractionation kit. Proteins in each fraction were analyzed by Western blotting as described in the legend for Figure 2C. D. HeLa cells were transfected with FLAG-tagged wild-type and E18A DJ-1 and treated with CCCP as described on the legend for Figure 5A. Seven hrs after CCCP treatment, cell extracts were treated with 100 µM DSS for 10 min and subjected to Western blot analysis with an anti-FLAG antibody.

It has been reported that some portions of DJ-1 are translocated into mitochondria when mitochondrial membrane potential is decreased [29], [33], [34]. To examine the effect of reduced mitochondrial membrane potential on the localization of E18A DJ-1, HeLa cells were transfected with wild-type DJ-1-HA or E18A DJ-1-HA. At 11 hrs after transfection, cells were treated with CCCP, an uncoupler of the oxidative phosphorylation system in mitochondria, for 7 hrs and treated with tetramethylrhodamine methyl ester (TMRM) for 15 min. The cells were then stained with anti-FLAG and anti-TOM20 antibodies. First, reduced mitochondrial membrane potential after treatment of cells with CCCP was confirmed by staining cells with TMRM, an indicator of mitochondrial membrane potential (Fig. 5B). While a portion of wild-type DJ-1-FLAG tended to be co-localized in mitochondria, a large portion of E18A DJ-1-FLAG was re-localized in the cytoplasm (Fig. 5A). Cell fractionation studies also showed that mitochondrial/membrane localization of E18A DJ-1-FLAG was reduced after CCCP treatment of cells (from 74.2 to 49.6) (Fig. 5C). To examine dimer formation of E18A DJ-1-FLAG in CCCP-treated cells, cross-linking experiments were carried out. The results showed that ratios of monomer and dimer E18A were reduced from 42.8 to 21.3 and increased from 57.2 to 68.7, respectively, after E18A-transfected cells were treated with CCCP (Fig. 5D).

### N-terminal Sequence is Necessary for Mitochondrial Localization of Mutants of DJ-1

**Figure 6:**
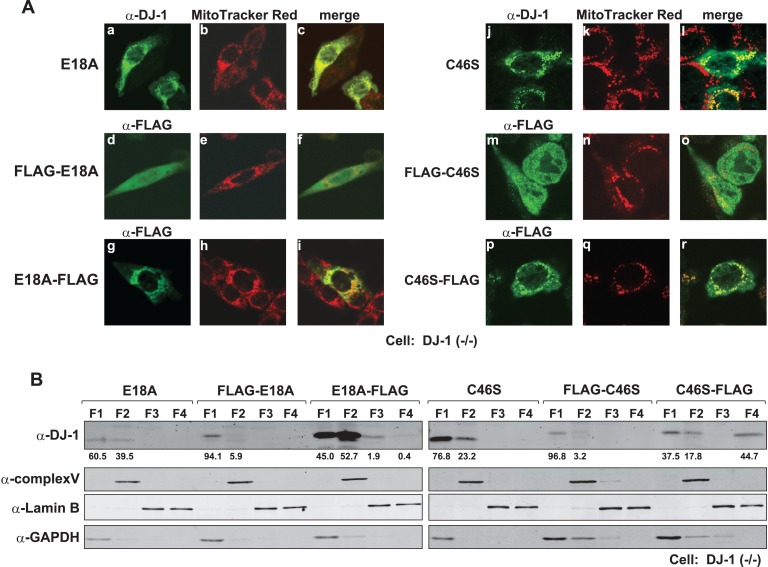
Effect of the tag position on localization of DJ-1 mutants. A. DJ-1(−/−) cells were transfected with tag-free E18A and C46S DJ-1 or with E18A and C46S DJ-1 containing an N- or C-terminal FLAG tag. At 24 hrs after transfection, the cells were fixed with 4% paraformaldehyde and with 0.1% TritonX-100 and stained with anti-FLAG and anti-DJ-1 antibodies. Cell images were then observed under a confocal laser microscope. B. DJ-1(−/−) cells that had been transfected as described in the legends for Figure 6A were fractionated by using a subcellular fractionation kit. Proteins in each fraction were analyzed by Western blotting as described in the legend for Figure 2C.

To examine the effects of N- and C-terminal regions of DJ-1 on its localization in cells, DJ-1(−/−) cells were transfected with E18A and C46S DJ-1 tagged with FLAG at the N-terminus or C-terminus and stained with an anti-FLAG antibody. Tag-free E18A and C46S DJ-1 were transfected as controls and stained with an anti-DJ-1 antibody, and mitochondria were stained with MitoTracker Red (Fig. 6). Tag-free E18A and C46S DJ-1 were located in mitochondria (Figs. 6A-a and 6A-j) as in the case of E18A and C46S DJ-1 with a C-terminal FLAG (Figs. 2, 3, 6A-g and 6A-p). E18A and C46S DJ-1 with an N-terminal FLAG were, on the other hand, found to be localized mainly in the cytoplasm (Figs. 6A-d and 6A-m). Cell fractionation studies using transfected cells clearly showed this correlation (Fig. 6B). These results suggest that proper mitochondrial localization of E18 and C46S DJ-1 is inhibited by addition of a FLAG sequence to the N-terminus of DJ-1.

**Figure 7:**
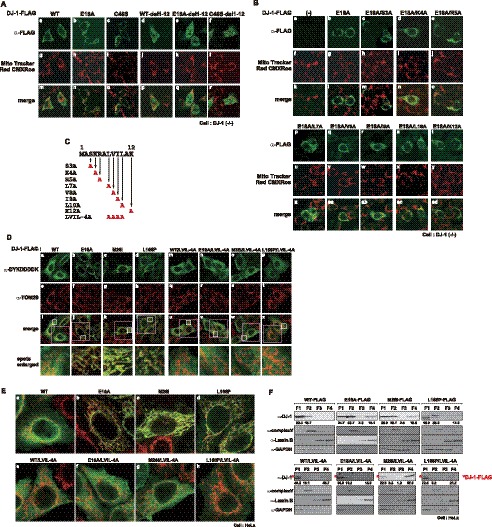
Identification of N-terminal sequence necessary for mitochondrial localization of mutant DJ-1. A. DJ-1(−/−) cells were transfected with wild-type, E18A, C46S DJ-1 and their mutants with deletion of the N-terminal 12 amino acids. At 24 hrs after transfection, the cells were treated with 100 nM MitoTracker Red for 45 min. After cells had been cultured without MitoTracker Red for 2 hrs, the cells were fixed with 4% paraformaldehyde and with acetone/methanol and stained with anti-FLAG and anti-DJ-1 antibodies. Cell images were then observed under a confocal laser microscope. B. DJ-1(−/−) cells were transfected with FLAG-tagged E18A DJ-1 containing an additional substitution mutation shown in Figure 7C. The cells were then subjected to immunostaining as described in the legend of Figure 7A. C. Schematic drawing of substitution mutants of DJ-1. D. HeLa cells were transfected with FLAG-tagged wild-type, E18A, M26I, L166P DJ-1 and their substation mutants shown in Figure 7C. The cells were then subjected to immunostaining with anti-DYKDDDDK and anti-TOM20 antibodies as described in the legend of Figure 7A. E. HeLa cells were transfected and were then subjected to immunostaining as described in the legends for Figure 7D. Cell images were enlarged. F. HeLa cells that had been transfected as described in the legends for Figure 7D were fractionated by using a subcellular fractionation kit. Proteins in each fraction were analyzed by Western blotting as described in the legend for Figure 2C.

DJ-1 consists of 9 α-helices and 6 β-sheets, and α-helix 1 is comprised of N-terminal 12 amino acids [47]. To examine the effect of the N-terminal sequence on localization of DJ-1 in cells, FLAG-tagged wild-type, E18A and C46S DJ-1 lacking the N-terminal 12 amino acids were transfected into DJ-1(−/−) cells and their localization was analyzed by staining cells with an anti-FLAG antibody (Fig. 7A). While mainly cytoplasmic localization of wild-type DJ-1-FLAG was a little changed by its N-terminal deletion, both E18A and C106S-FLAG were translocated from mitochondria to the cytoplasm (Figs. 7A e-f), suggesting that the N-terminal 12 amino acids are required for E18A and C46S DJ-1 to be localized in mitochondria. To identify the amino acid(s) specific to determination of mitochondrial localization, each amino acid at numbers 3–12 in E18A DJ-1 was substituted to A (Fig. 7C) and localization of 7 substituted mutants of E18A DJ-1-FLAG was examined after transfection into DJ-1(−/−) cells. Localization of the substituted mutants of E18A DJ-1-FLAG tested was not changed, and they remained in mitochondria (Fig. 7B). Then leucine, valine, isoleucine and leucine at amino acid numbers 7, 8, 9 and 10, respectively, in E18A DJ-1 were changed to A. As shown in Fig. 7D-n, this mutant (E18A/LVIL-4A) of E18A DJ-1-FLAG was localized in the cytoplasm. The other mitochondrial localized mutants, M26I and L166P DJ-1, were also re-localized from mitochondria to the cytoplasm by this substitution (Figs. 7D-o and -p). A small portion of wild-type DJ-1s harboring these substitution mutations tended to change their localization compared to that of wild-type DJ-1 (Figs. 7D-a and 7D-m). When merged cell images were enlarged, clear localization changes of DJ-1 were observed (Fig. 7E). Mitochondrial localization of E18A, M26I and L166P DJ-1-FLAG was inhibited by alanine substitution and some portion of wild-type DJ-1-FLAG tended to be re-localized. Furthermore, cell fractionation studies clearly showed that a large portion of E18A, M26I and L166P DJ-1 (around 50% to less than 10%) and small portion of wild-type DJ-1 (from 16.7% to 10.1%) were re-localized from mitochondria to cytoplasm and cytoskeleton fractions (Fig. 7F). These results suggest that the N-terminal 12 amino acids, at least leucine, valine, isoleucine and leucine, are necessary for mitochondrial localization of DJ-1 mutants that do not form a homodimer.

## Discussion

In this study, we found that unlike C106S DJ-1, E18 mutants of DJ-1 are oxidized in a manner similar to that of wild-type DJ-1. We then found that E18 mutants of DJ-1 do not make homodimers and are localized in mitochondria. These phenomena are true for pathogenic mutants, M26I and L166P DJ-1, and for dimer formation-negative mutants, C46S and C56A DJ-1. Furthermore, the N-terminal 12 amino acids were found to be necessary for mitochondrial localization of these mutants of DJ-1. These findings indicate that E18 mutants of DJ-1 are useful tools for analyzing DJ-1′s properties. Furthermore, monomer DJ-1 was found to be localized in mitochondria.

The catalytic triad in DJ-1 is comprised of E18, H126 and C106 [47], and integrity of C106 is essential for all of the functions of DJ-1 [3], [15], [17]. It has been reported that formation of a sulfinic acid of C106 through hydrogen bonding to E18 is critical for mitochondrial function of DJ-1 and that various E18 mutants differentially affect oxidation of C106 [45]. When we compare our localization results of E18 mutants with those of Blackinton et al. [45], there is a slight difference: E18D DJ-1, like C106A DJ-1, is localized mainly in the cytoplasm in M17 neuroblastoma cells [45], but E18D DJ-1 was found to be localized in the cytoplasm and mitochondria in DJ-1(−/−) cells in this study (Fig. 2E). This difference might be due to different cells used. Of E18 mutants of DJ-1, E18D and E18S mutants showed three localization patterns, in the cytoplasm, mitochondrial and both. Immunoprecipitation and pull-down experiments showed that both mutants did not form stable dimer and that the level of dimer formation of E18D and E18S mutants was similar to that of other E18 mutants, suggesting that E18D and E18S DJ-1 are present in the cytoplasm as a monomer due to insufficient import into mitochondria. It is therefore thought that interaction of E18D and E18S DJ-1 with protein(s) recruiting DJ-1 into mitochondrial is reduced. Alternatively, these mutations may change interaction of DJ-1 with cytoplasmic protein(s) that retains DJ-1 in cytoplasm. Since the N-terminal region of DJ-1 is necessary for translocation of E18A DJ-1 in mitochondria and since E18 is located close to the N-terminal region, however, it is likely that mutations of E18 affect the recruiting process of DJ-1 into mitochondria. A previous studies showed that oxidation of C106 as a sulfinic acid is required for mitochondrial localization of DJ-1 [3] and that a side chain of E18 is connected with C106 by a hydrogen bond that influences its pKa [46], the results in this study showed that E18A/C106S DJ-1, in which oxidation of C106 does not occur, was localized in mitochondria (Fig. 2B) and that oxidation of C106 is not necessary for dimer formation (Fig. 1), indicating that oxidation of C106 is required but not essential for mitochondrial localization of DJ-1. These results also suggest that integrity of the structure around E18 and C106 is important for mitochondrial localization of DJ-1 and that the side chain of E18 has a role in discrimination and interaction of protein(s) that regulates localization of DJ-1. Ratios of mitochondrial localization of DJ-1 were a little changed by immunofluorescence and by subcellular fractionation techniques. It would be explained as follows: Fixation of cells with 0.2% Triton X-100 in immunofluorescence experiments tends to release a small portion of cytoplasmic proteins from cells. Subcellular fractionation of proteins, on the other hand, retains all of the proteins in respective fractions. The different amount of cytoplasmic proteins in two methods leads to small difference of ratios of mitochondrial localization of DJ-1.

It has been reported that mitochondria-localized DJ-1, including pathogenic M26I and L166P DJ-1, is not able to form a dimer [2], [48]. Dimer formation is necessary for DJ-1 to elicit its function [15], and anti-oxidative stress activities of M26I and L166P DJ-1 and E18 mutants of DJ-1 are weaker than that of wild-type DJ-1 [45], [49]. Anti-oxidative stress activity of DJ-1 tagged with a mitochondrial target sequence is stronger than that of wild-type DJ-1 [27], and some portions of DJ-1 are translocated into mitochondria after cells receive oxidative stress [3], [35]. Considering these findings, it is thought that localization of DJ-1 as a dimer in mitochondria is required for DJ-1 to play a role in anti-oxidative stress reaction and that DJ-1 localized in mitochondria as a monomer, such as M26I and L166P DJ-1, is, in contrast, harmful to cells.

Since DJ-1 contains no mitochondrial targeting signal, it is not known how DJ-1 is re-localized in mitochondria. We found that deletion or mutation of the N-terminal 12 amino acids in DJ-1 resulted in location of E18A, M26I and L166P DJ-1 in the cytoplasm (Fig. 7). When green fluorescence protein (GFP) was linked to the N-terminal 12 amino acids of DJ-1 and transfected into cells, however, GFP was not translocated into mitochondria and was located in whole cells (data not shown), suggesting that the N-terminal 12-amino acid sequence of DJ-1 is not a bona fide mitochondrial targeting signal but that the N-terminal 12 amino acids are necessary for monomer DJ-1 to be localized in mitochondria.

When mitochondrial membrane potential was reduced after cells had been treated with CCCP, some portions of wild-type DJ-1 tended to be translocated to mitochondria as reported previously [29], [31], [34]. E18A DJ-1 was, on the other hand, localized in the cytoplasm (Fig. 5). Reduction of mitochondrial membrane potential occurs when cells receive oxidative stress, and DJ-1 translocated into mitochondria may induce mitophagy to degrade damaged mitochondria, thereby maintaining mitochondrial homeostasis (see a recent review [50]). By using two permeabilization methods, E18A, M26I and L166P DJ-1 were found to be localized inside the outer mitochondrial membrane, including the inner membrane and matrix (Fig. 4). Important machineries such as oxidative phosphorylation and glycolytic systems are present in the inner membrane and matrix. Although it is not clear why E18A DJ-1 is re-localized from mitochondria into the cytoplasm under the condition of reduced mitochondrial membrane potential, it is thought that monomer DJ-1 is easily shuttled between compromised mitochondria and the cytoplasm and that cells exclude monomer DJ-1, which is harmful DJ-1, from mitochondria in order to prevent further damage to mitochondria. Alternatively, it is also thought that E18A DJ-1 is not able to be imported into mitochondria due to reduced activity of the import system under the condition of reduced mitochondrial membrane potential. Further experiments are necessary to assess these possibilities.

## References

[1] Nagakubo D, Taira T, Kitaura H, Ikeda M, Tamai K, et al (1997) DJ-1, a novel oncogene which transforms mouse NIH3T3 cells in cooperation with ras. Biochem Biophys Res Commum 231: 509–513. 10.1006/bbrc.1997.6132 9070310

[2] Bonifati V, Rizzu P, van Baren MJ, Schaap O, Breedveld GJ, et al (2003) Mutations in the DJ-1 gene associated with autosomal recessive early-onset Parkinsonism. Science 299: 256–259. 12446870 10.1126/science.1077209

[3] Canet-Aviles RM, Wilson MA, Miller DW, Ahmad R, McLendon C, et al (2004) The Parkinson’s disease protein DJ-1 is neuroprotective due to cysteine-sulfinic acid-driven mitochondrial localization. Proc Natl Acad Sci USA 101: 9103–9108. 15181200 10.1073/pnas.0402959101 PMC428480

[4] Zhang L, Shimoji M, Thomas B, Moore DJ, Yu S, et al (2005) Mitochondrial localization of the Parkinson’s disease related protein DJ-1: implications for pathogenesis. Hum Mol Genet 14: 2063–2073. 15944198 10.1093/hmg/ddi211

[5] Shinbo Y, Niki T, Taira T, Ooe H, Takahashi-Niki K, et al (2006) Proper SUMO-1 conjugation is essential to DJ-1 to exert its full activities. Cell Death Diff 13: 96–108. 10.1038/sj.cdd.4401704 15976810

[6] Takahashi K, Taira T, Niki T, Seino C, Iguchi-Ariga SMM, et al (2001) DJ-1 positively regulates the androgen receptor by impairing the binding of PIASx to the receptor. J Biol Chem 276: 37556–37563. 11477070 10.1074/jbc.M101730200

[7] Niki T, Takahashi-Niki K, Taira T, Iguchi-Ariga SMM, Ariga H (2003) DJBP: A novel DJ-1-binding protein, negatively regulates the androgen receptor by recruiting histone deacetylase complex, and DJ-1 antagonizes this inhibition by abrogation of this complex. Mol Cancer Res 1: 247–261. 12612053

[8] Shinbo Y, Taira T, Niki T, Iguchi-Ariga SMM, Ariga H (2005) DJ-1 restores p53 transcription activity inhibited by Topors/p53BP3. Int J Oncol 26: 641–648. 15703819

[9] Zhong N, Kim CY, Rizzu P, Geula C, Porter DR, et al (2006) DJ-1 transcriptionally up-regulates the human tyrosine hydroxylase by inhibiting the sumoylation of pyrimidine tract-binding protein-associated splicing factor. J Biol Chem 281: 20940–20948. 16731528 10.1074/jbc.M601935200

[10] Clements CM, McNally RS, Conti BJ, Mak TW, Ting JP (2006) DJ-1, a cancer- and Parkinson’s disease-associated protein, stabilizes the antioxidant transcriptional master regulator Nrf2. Proc Natl Acad Sci USA 103: 15091–15096. 17015834 10.1073/pnas.0607260103 PMC1586179

[11] Tillman JE, Yuan J, Gu G, Fazli L, Ghosh R, et al (2007) DJ-1 binds androgen receptor directly and mediates its activity in hormonally treated prostate cancer cells. Cancer Res 67: 4630–4637. 17510388 10.1158/0008-5472.CAN-06-4556

[12] Ishikawa S, Taira T, Niki T, Takahashi-Niki K, Maita C, et al (2009) Oxidative status of DJ-1-dependent activation of dopamine synthesis through interaction of tyrosine hydroxylase and 4-dihydroxy-L-phenylalanine (L-DOPA) decarboxylase with DJ-1. J Biol Chem 284: 28832–28844. 19703902 10.1074/jbc.M109.019950 PMC2781429

[13] Fan J, Ren H, Ji N, Fei E, Zhou T, et al (2008) DJ-1 decreases bax expression through repressing p53 transcriptional activity. J Biol Chem 283: 4022–4030. 18042550 10.1074/jbc.M707176200

[14] Xu J, Zhong N, Wang H, Elias JE, Kim CY, et al (2005) The Parkinson’s disease-associated DJ-1 protein is a transcriptional co-activator that protects against neuronal apoptosis. Hum Mol Genet 14: 1231–1241. 15790595 10.1093/hmg/ddi134

[15] Taira T, Saito Y, Niki T, Iguchi-Ariga SMM, Takahashi K, et al (2004) DJ-1 has a role in antioxidative stress to prevent cell death. EMBO Rep 5: 213–218. 14749723 10.1038/sj.embor.7400074 PMC1298985

[16] Kinumi T, Kimata J, Taira T, Ariga H, Niki E (2004) Cysteine-106 of DJ-1 is the most sensitive cysteine residue to hydrogen peroxide-mediated oxidation in vivo in human umbilical vein endothelial cells. Biochem Biophys Res Commun 317: 722–728. 15081400 10.1016/j.bbrc.2004.03.110

[17] Martinat C, Shendelman S, Jonason A, Leete T, Beal MF, et al (2004) Sensitivity to oxidative stress in DJ-1-deficient dopamine neurons: An ES derived cell model of primary Parkinsonism. PLoS Biol 2: e327. 15502868 10.1371/journal.pbio.0020327 PMC521171

[18] Inden M, Taira T, Kitamura Y, Yanagida T, Tsuchiya D, et al (2006) PARK7 DJ-1 protects against degeneration of nigral dopaminergic neurons in Parkinson’s disease rat model. Neurobiol Dis 24: 144–158. 16860563 10.1016/j.nbd.2006.06.004

[19] Yanagida T, Tsushima J, Kitamura Y, Yanagisawa D, Takata K, et al (2009) Oxidative stress induction of DJ-1 protein in reactive astrocytes scavenges free radicals and reduces cell injury. Oxid Med Cell Longev 2: 36–42. 20046643 10.4161/oxim.2.1.7985 PMC2763229

[20] Shendelman S, Jonason A, Martinat C, Leete T, Abeliovich A (2004) DJ-1 is a redox-dependent molecular chaperone that inhibits alpha-synuclein aggregate formation. PLoS Biol 2: e362. 15502874 10.1371/journal.pbio.0020362 PMC521177

[21] Zhou W, Zhu M, Wilson MA, Petsko GA, Fink AL (2006) The oxidation state of DJ-1 regulates its chaperone activity toward α-synuclein. J Mol Biol 356: 1036–1048. 16403519 10.1016/j.jmb.2005.12.030

[22] Olzmann JA, Brown K, Wilkinson KD, Rees HD, Huai Q, et al (2004) Familial Parkinson’s disease-associated L166P mutation disrupts DJ-1 protein folding and function. J Biol Chem 279: 8506–8515. 14665635 10.1074/jbc.M311017200

[23] Koide-Yoshida S, Niki T, Ueda M, Himeno S, Taira T, et al (2007) DJ-1 degrades transthyretin and an inactive form of DJ-1 is secreted in familial amyloidotic polyneuropathy. Int J Mol Med 19: 885–893. 17487420

[24] Chen J, Li L, Chin LS (2010) Parkinson disease protein DJ-1 converts from a zymogen to a protease by carboxyl-terminal cleavage. Hum Mol Genet 19: 2395–408. 20304780 10.1093/hmg/ddq113 PMC2876885

[25] Ooe H, Taira T, Iguchi-Ariga SMM, Ariga H (2005) Induction of reactive oxygen species by bisphenol A and abrogation of bisphenol A-induced cell injury by DJ-1. Toxicol Sci 88: 114–126. 16093527 10.1093/toxsci/kfi278

[26] Li HM, Niki T, Taira T, Iguchi-Ariga SMM, Ariga H (2005) Association of DJ-1 with chaperones and enhanced association and colocalization with mitochondrial Hsp70 by oxidative stress. Free Radic Res 39: 1091–1099. 16298734 10.1080/10715760500260348

[27] Junn E, Jang WE, Zhao X, Jeong B, Mouradian MM (2009) Mitochondrial localization of DJ-1 leads to enhanced neuroprotection. J Neurosci Res 87: 123–129. 18711745 10.1002/jnr.21831 PMC2752655

[28] Hayashi T, Ishimori C, Takahashi-Niki K, Taira T, Kim YC, et al (2009) DJ-1 binds to mitochondrial complex I and maintains its activity. Biochem Biophys Res Commun 390: 667–672. 19822128 10.1016/j.bbrc.2009.10.025

[29] Heo JY, Park JH, Kim SJ, Seo KS, Han JS, et al (2012) DJ-1 null dopaminergic neuronal cells exhibit defects in mitochondrial function and structure: involvement of mitochondrial complex I assembly. PLoS One 7: e32629. 22403686 10.1371/journal.pone.0032629 PMC3293835

[30] Ren H, Fu K, Wang D, Mu C, Wang G (2011) Oxidized DJ-1 interacts with the mitochondrial protein BCL-XL. J Biol Chem 286: 35308–35317. 21852238 10.1074/jbc.M110.207134 PMC3186373

[31] Thomas KJ, McCoy MK, Blackinton J, Beilina A, van der Brug M, et al (2011) DJ-1 acts in parallel to the PINK1/parkin pathway to control mitochondrial function and autophagy. Hum Mol Genet 20: 40–50. 20940149 10.1093/hmg/ddq430 PMC3000675

[32] Irrcher I, Aleyasin H, Seifert EL, Hewitt SJ, Chhabra S, et al (2010) Loss of the Parkinson’s disease-linked gene DJ-1 perturbs mitochondrial dynamics. Hum Mol Genet 19: 3734–3746. 20639397 10.1093/hmg/ddq288

[33] Hao LY, Giasson BI, Bonini NM (2010) DJ-1 is critical for mitochondrial function and rescues PINK1 loss of function. Proc Natl Acad Sci USA 107: 9747–9752. 20457924 10.1073/pnas.0911175107 PMC2906840

[34] Krebiehl G, Ruckerbauer S, Burbulla LF, Kieper N, Maurer B, et al (2010) Reduced basal autophagy and impaired mitochondrial dynamics due to loss of Parkinson’s disease-associated protein DJ-1. PLoS One 5: e9367. 20186336 10.1371/journal.pone.0009367 PMC2826413

[35] Wang X, Petrie TG, Liu Y, Liu J, Fujioka H, et al (2012) Parkinson’s disease-associated DJ-1 mutations impair mitochondrial dynamics and cause mitochondrial dysfunction. J Neurochem 121: 830–839. 22428580 10.1111/j.1471-4159.2012.07734.x PMC3740560

[36] Bandopadhyay R, Kingsbury AE, Cookson MR, Reid AR, Evans IM, et al (2004) The expression of DJ-1 (PARK7) in normal human CNS and idiopathic Parkinson’s disease. Brain 127: 420–430. 14662519 10.1093/brain/awh054

[37] Choi J, Sullards MC, Olzmann JA, Rees HD, Weintraub ST, et al (2006) Oxidative damage of DJ-1 is linked to sporadic Parkinson and Alzheimer diseases. J Biol Chem 281: 10816–10824. 16517609 10.1074/jbc.M509079200 PMC1850953

[38] Mizuno Y, Ohta S, Tanaka M, Takamiya S, Suzuki K, et al (1989) Deficiencies in complex I subunits of the respiratory chain in Parkinson’s disease. Biochem Biophys Res Commun 163: 1450–1455. 2551290 10.1016/0006-291x(89)91141-8

[39] Parker Jr WD, Boyson SJ, Parks JK (1989) Abnormalities of the electron transport chain in idiopathic Parkinson’s disease. Ann Neurol 26: 719–723. 2557792 10.1002/ana.410260606

[40] Schapira AH, Cooper JM, Dexter D, Clark JB, Jenner P, et al (1990) Mitochondrial complex I deficiency in Parkinson’s disease. J Neurochem 54: 823–827. 2154550 10.1111/j.1471-4159.1990.tb02325.x

[41] Orth M, Schapira AH (2002) Mitochondrial involvement in Parkinson’s disease. Neurochem Int 40: 533–541. 11850110 10.1016/s0197-0186(01)00124-3

[42] Mizuno Y, Hattori N, Matsumine H (1998) Neurochemical and neurogenetic correlates of Parkinson’s disease. J Neurochem 71: 893–902. 9721714 10.1046/j.1471-4159.1998.71030893.x

[43] Giaime E, Yamaguchi H, Gautier CA, Kitada T, Shen J (2012) Loss of DJ-1 does not affect mitochondrial respiration but increases ROS production and mitochondrial permeability transition pore opening. PLoS One 7: e40501. 22792356 10.1371/journal.pone.0040501 PMC3392228

[44] Kim YC, Kitaura H, Iguchi-Ariga SMM, Ariga H (2010) DJ-1, an oncogene and causative gene for familial Parkinson’s disease, is essential for SV40 transformation in mouse fibroblasts through up-regulation of c-Myc. FEBS Lett 584: 3891–3895. 20708612 10.1016/j.febslet.2010.08.010

[45] Blackinton J, Lakshminarasimhan M, Thomas KJ, Ahmad R, Greggio E, et al (2009) Formation of a stabilized cysteine sulfinic acid is critical for the mitochondrial function of the parkinsonism protein DJ-1. J Biol Chem 284: 6476–6485. 19124468 10.1074/jbc.M806599200 PMC2649108

[46] Witt AC, Lakshminarasimhan M, Remington BC, Hasim S, Pozharski E, et al (2008) Cysteine pKa depression by a protonated glutamic acid in human DJ-1. Biochemistry 47: 7430–7440. 18570440 10.1021/bi800282d PMC2760839

[47] Honbou K, Suzuki NN, Horiuchi M, Niki T, Taira T, et al (2003) The crystal structure of DJ-1, a protein related to male fertility and Parkinson’s disease. J Biol Chem 278: 31380–31384. 12796482 10.1074/jbc.M305878200

[48] Hulleman JD, Mirzaei H, Guigard E, Taylor KL, Ray SS, et al (2007) Destabilization of DJ-1 by familial substitution and oxidative modifications: implications for Parkinson’s disease. Biochemistry 46: 5776–5789. 17451229 10.1021/bi7001778

[49] Takahashi-Niki K, Niki T, Taira T, Iguchi-Ariga SMM, Ariga H (2004) Reduced anti-oxidative stress activities of DJ-1 mutants found in Parkinson’s disease patients. Biochem Biophys Res Commun 320: 389–397. 15219840 10.1016/j.bbrc.2004.05.187

[50] McCoy MK, Cookson MR (2011) DJ-1 regulation of mitochondrial function and autophagy through oxidative stress. Autophagy 7: 531–532. 21317550 10.4161/auto.7.5.14684 PMC3127213

